# A new species of *Phyllocomus* Grube, 1878 from the Yellow Sea, China (Annelida, Ampharetidae)

**DOI:** 10.3897/zookeys.676.11828

**Published:** 2017-05-23

**Authors:** Jixing Sui, Xinzheng Li

**Affiliations:** 1 Institute of Oceanology, Chinese Academy of Sciences, Qingdao 266071, China; 2 Laboratory for Marine Biology and Biotechnology, Qingdao National Laboratory for Marine Science and Technology, Qingdao 266071, China; 3 University of Chinese Academy of Sciences, Beijing 100049, China

**Keywords:** Polychaete, *Phyllocomus chinensis* sp. n., *Schistocomus*, taxonomy

## Abstract

A new species of the ampharetid genus *Phyllocomus*, *P. chinensis* **sp. n.**, is described based on material from the Yellow Sea. The new species is distinguished from the known species of this genus by having two thoracic regions, with segments of the anterior region (up to thoracic chaetiger 10) approximately half as long as those of the posterior region, neuropodia of the anterior region are large while those of the posterior region become gradually smaller, thoracic neuropodia without dorsal cirrus, and abdominal neuropodia with a papillary dorsal cirrus. A key to all species of *Phyllocomus* is given.

## Introduction

Ampharetids are small to medium-sized, tubiculous worms which have a worldwide marine distribution from the intertidal to 8292 m deep (Jirkov 2011; Jumars et al. 2015). Ampharetidae comprise approximately 230 species distributed among 62 genera, 34 of them monotypic (Read 2017). The genus *Phyllocomus* was erected in 1878 by Grube for the species *P. crocea* Grube, 1878. Holthe (2000) described the species *P. balinensis* Holthe, 2000 and characterized the genus *Phyllocomus* as having four pairs of branchiae, at least two of these foliate, twelve thoracic uncinigers and a long abdomen.

The genus *Schistocomus* Chamberlin, 1919 resembles *Phyllocomus* in having four pairs of branchiae, twelve thoracic uncinigers, and a long abdomen. It differs from the latter in having branchiae of two types, one pair smooth and awl-shaped, and the other three with one or two series of lamellar branches. However, in *Phyllocomus* the two known species also both have two types of branchiae, awl-shaped and foliate. So, we agree with Day (1964) that *Schistocomus* is a synonym of *Phyllocomus*. Although Reuscher et al. (2009) considered that *Schistocomus* was a valid genus, he now agrees with Day (1964) (pers. comm. Reuscher, 2016). Thus, five valid species have been described in the genus *Phyllocomus*: *P. crocea* Grube, 1878 from the Southern Ocean; *P. balinensis* Holthe, 2000 from the Bali Sea; *P. hiltoni* (Chamberlin, 1919) from Laguna Beach (USA); *P. fauveli* (Hartman, 1955) from India; and *P. sovjeticus* (Annenkova, 1937) from the Japanese Sea.

Recently, two *Phyllocomus* specimens were identified and separated during sorting of material of Ampharetidae deposited in the Marine Biological Museum of the Chinese Academy of Sciences (MBMCAS). These specimens represent an undescribed species. They are described herein and proposed as a new species to science.

## Materials and methods

The two specimens were collected using a 1.5×0.5 m Agassiz trawl from the Yellow Sea by the team investigating a project entitled “The key processes, mechanism and ecological consequences of jellyfish blooms in China coastal waters” in June 2012 (Qiu, 2014). They were fixed in ethanol and preserved in 75% ethanol. The specimens are deposited in the Marine Biological Museum of the Chinese Academy of Sciences (**MBMCAS**). The specimens were photographed with a digital camera attached to a Nikon AZ100 microscope and drawn with camera lucida attached to a Nikon SMZ1500 microscope.

## Systematics

### Family Ampharetidae Malmgren, 1866

#### Genus *Phyllocomus* Grube, 1878

##### Phyllocomus chinensis sp. n.

Taxon classificationAnimaliaAnnelidaAmpharetidae

http://zoobank.org/ECA63BE1-2F58-4AB2-BA5F-A84D754E2F98

Figs 1 , 2 , 3

###### Type material.

**Holotype**: complete. MBM285071. Yellow Sea, Station A3 (36°59'28"N, 123°58'17"E); depth 77 m; shell and sand; coll. Dong, D. and Sui J.; 28 June 2012.

###### Paratype.

complete. MBM285072, same locality.

###### Diagnosis.

Prostomium with two rows of eyes, approximately ten in each row, appear to be crescent-shaped. Buccal tentacles smooth. Paleae and postbranchial hooks absent. Four pairs of branchiae. Twelve thoracic uncinigerous segments, 34 abdominal uncinigerous segments, without rudimentary notopodia. Pygidium with two pairs of long cirri.

###### Description.

**Figure 1.**
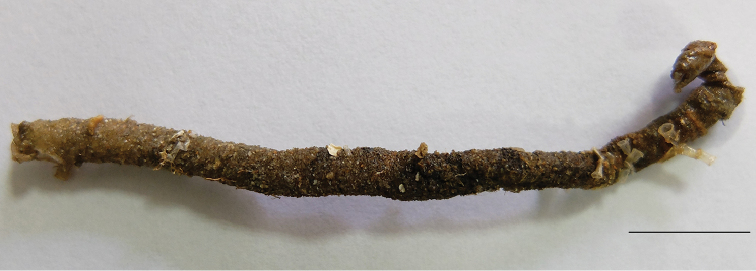
*Phyllocomus chinensis* sp. n., tube of holotype. Scale bar 2 cm.

**Figure 2.**
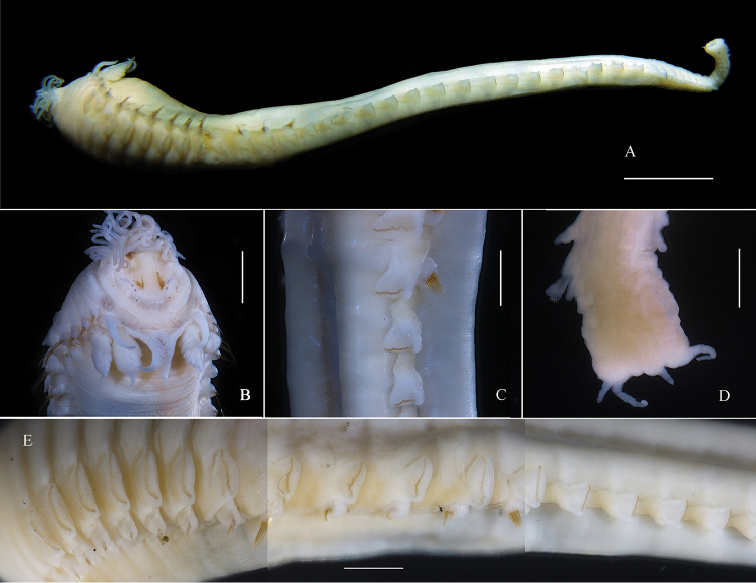
*Phyllocomus chinensis* sp. n. **A** whole specimen, lateral view **B** anterior end, dorsal view **C** last thoracic and first abdominal segments, lateral view **D** posterior region (with two pairs of long anal cirri) **E** consecutive variation of the neuropodia from segment 6 to segment 21. Scale bars **A**: 4 mm, **B, E**: 2mm, **C–D**: 1 mm.

Holotype. Tube cylindrical, black, with broken shells and sand (Fig. 1). Length 36 mm, thorax width 5 mm without chaetae. Thorax and abdomen well defined; thorax approximately twice width of abdomen (Fig. 2A). Color in alcohol pale yellow; appear to be some pigmentation on prostomium.

**Figure 3.**
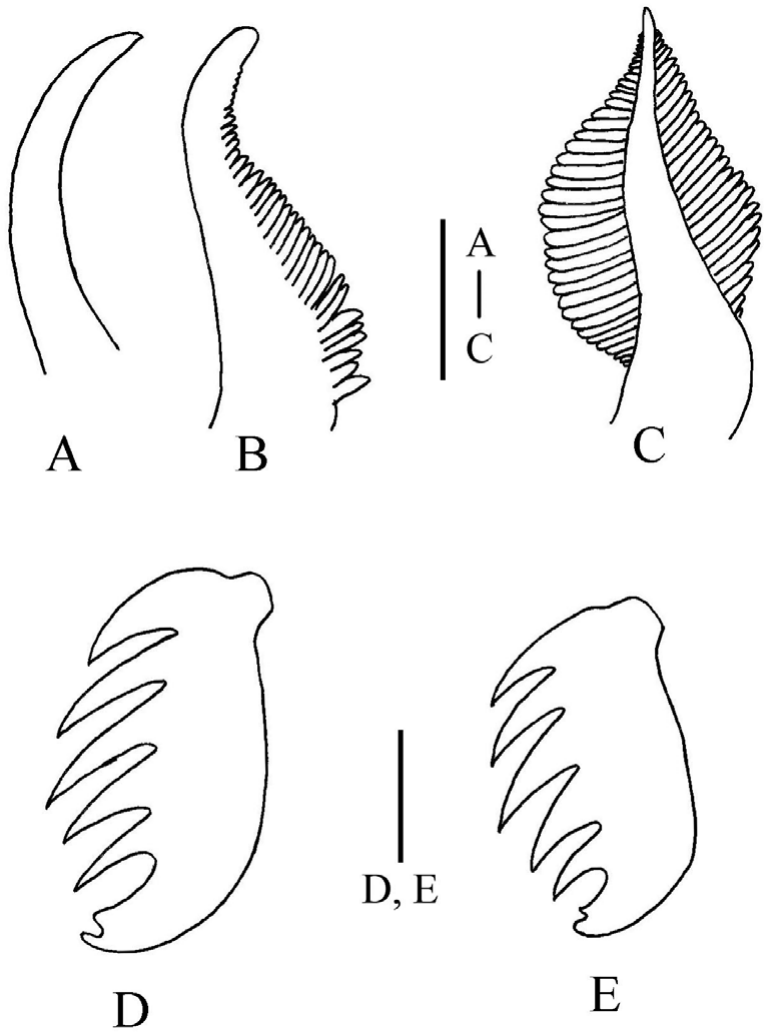
*Phyllocomus chinensis* sp. n. **A** awl-shaped branchiae from segment III **B** branchiae with one row of lamellae from segment II **C** branchiae with two rows of lamellae from segment 5 **D** thoracic uncinusfrom segment 7, lateral view; **E** abdominal uncinus from segment 20, lateral view. Scale bars **A–C**: 1 mm, **D–E**: 10 µm.

Prostomium feebly developed on dorsum and forming lower triangular lobe ventrally with convex anterior margin. Two rows of eyes, approximately ten in each row, appear to be crescent-shaped. Buccal tentacles smooth (Fig. 2B). First segment achaetous. Paleae and postbranchial hooks absent. Four pairs branchiae. Innermost branchiae of anterior transverse row originating from segment II, outermost branchiae of anterior transverse row originating from segment III, outer pair awl-shaped, smooth (Fig. 3A), inner pair with single series of pectinate lamellae (Fig. 3B). Innermost branchiae of posterior transverse row originating from segment IV, outermost branchiae of posterior transverse row originating from segment V, two pairs of branchiae both with double rows of lamellae (Fig. 3C).

Notopodia begin on segment III, present in 15 segments. Notopodia well-developed, conical, bearing bundle of winged capillary chaetae. Notopodia and capillaries of third to fifth segments increasing gradually in size. Neuropodial uncini begin on fourth chaetiger (segment VI) and present in 12 thoracic segments. Thorax sharply subdivided into two regions. Segments of anterior region (up to thoracic chaetiger 10) approximately half as long as those of posterior region, neuropodia of anterior region large, and similar-sized, while those of posterior region become gradually smaller; the neuropodia of last thoracic unciniger is half size of first thoracic unciniger. Neuropodia of thoracic uncinigers are tori, without dorsal cirrus; neuropodia of abdominal uncinigerous are pinnules, with papillary dorsal cirrus (Fig. 2E). Continuous ventral shields present to approximately thoracic unciniger 7. Elevated or modified notopodia absent. Thirty-four abdominal uncinigerous segments, without rudimentary notopodia (Fig. 2C). Thoracic torus 1 mm long, with approximately 68 uncini. Abdominal torus 0.5 mm long, with approximately 38 uncini. Uncini in abdominal segments are smaller than those of thorax. All uncini with single row of five teeth (Fig. 3D, E). Pygidium with two pairs of long cirri (Fig. 2D).

###### Variation.

Paratype 25 mm long, 4 mm wide without chaetae, has 35 abdominal uncinigerous segments.

###### Etymology.

The species is named after its type locality on the coast of China. The species name is an adjective in the nominative singular, derived from China, with the Latin suffix -ensis to indicate the Chinese seas.

###### Distribution.

Yellow Sea at 77m depth. It is suspected that some species-list records of *P. hiltoni* and *P. sovjeticus* from China belong to *P. chinensis* sp. n. (Huang 1994; Liu 2008). Examination of more material from different localities will establish a more accurate distribution of the new species.

###### Remarks.

Three species of *Phyllocomus*, *P. hiltoni* (Chamberlin, 1919), *P. fauveli* (Hartman, 1955) and *P. sovjeticus* (Annenkova, 1937), are similar to the new species. They all have branchiae of the same type. *Phyllocomus hiltoni* and *P. fauveli* differ from the new species by having a long dorsal cirrus in the abdominal neuropodium, while the new species has a papillary dorsal cirrus. There are two major differences between the new species and *P. sovjeticus*: (1) the new species has thoracic neuropodia without dorsal cirri, while *P. sovjeticus* has large rounded, feebly-distinct papillary dorsal cirri (Annenkova 1937), (2) the new species has abdominal segments without rudimentary notopodia, while *P. sovjeticus* has a small and rounded rudimentary lobe (Annenkova 1937). Both are important characters to distinguish ampharetids species. Otherwise, the new species has two rows of eyes, approximately ten in each row, which appear to be crescent-shaped, 34–35 abdominal uncinigerous segments, and two pairs of long cirri in the pygidium, while the latter has no eyes, 44-54 abdominal uncinigerous segments, and a few rounded papillae on the pygidium (Okuda 1947). A key to all species of *Phyllocomus* is provided below.

#### Key to *Phyllocomus* species

**Table:** 

1	At least two pairs foliate branchiae	**2**
–	Three of 4 pairs of lamellate branchiae	**3**
2	Bases of last pair of branchiae as long as remaining branchial bases	***P. crocea* Grube, 1878**
–	Bases of last pair of branchiae more than twice as long as remaining branchial bases	***P. balinensis* Holthe, 2000**
3	Abdominal neuropodia with long dorsal cirri	**4**
–	Abdominal neuropodia with papillary dorsal cirri	**5**
4	Awl-shaped and unipinnate pairs of branchiae in one transverse row	***P. fauveli* (Hartman, 1955)**
–	Unipinnate pair of branchiae located in front of awl-shaped pair	***P. hiltoni* (Chamberlin, 1919)**
5	Thoracic neuropodia without dorsal cirri	***P. chinensis* sp. n.**
–	Thoracic neuropodia with large papillary dorsal cirri	***P. sovjeticus* (Annenkova, 1937)**

## Supplementary Material

XML Treatment for Phyllocomus chinensis
